# Hand Laser Perfusion Imaging to Assess Radial Artery Patency: A Pilot Study

**DOI:** 10.3390/jcm7100319

**Published:** 2018-10-02

**Authors:** Ciro Indolfi, Francesco Passafaro, Sabato Sorrentino, Carmen Spaccarotella, Annalisa Mongiardo, Daniele Torella, Alberto Polimeni, Jolanda Sabatino, Antonio Curcio, Salvatore De Rosa

**Affiliations:** 1 Division of Cardiology, Department of Medical and Surgical Sciences, Magna Graecia University, 88100 Catanzaro, Italy; passafaro.francesco@gmail.com (F.P.); sabatosorrentino@hotmail.com (S.S.); c.spaccarotella@iol.it (C.S.); amongiardo@tin.it (A.M.); dtorella@unicz.it (D.T.); albj_13@hotmail.com (A.P.); jolesbt@hotmail.it (J.S.); curcio@unicz.it (A.C.); saderosa@unicz.it (S.D.R.); 2 URT-CNR, Magna Graecia University, 88100 Catanzaro, Italy

**Keywords:** radial artery occlusion, Laser Doppler, access site complication, PCI, coronary angiography

## Abstract

Objectives: To test a novel diagnostic technique to assess radial artery perfusion after transradial catheterization. Background: Despite being mostly asymptomatic, radial artery occlusion (RAO) is not a benign complication, and its diagnosis is frequently missed because it requires time-consuming diagnostic testing. We developed a novel operator-independent diagnostic test to assess RAO after coronary procedures through a transradial access (TRA) by means of hand Laser Perfusion Imaging (LPI). Methods: One hundred patients were evaluated before and after TRA by means of the LPI. A radial perfusion index (RPI) was calculated as the ratio between the total perfusion measured during ulnar occlusion and total basal perfusion. Vascular Duplex scan (VDS) was used as the standard of reference to assess the artery patency. Results: LPI correctly identified RAO in 100% of cases. Post-procedural RPI was 0.89 ± 0.13 in patients with radial patency vs. 0.15 ± 0.04 in patients with RAO (*p* < 0.001). In line with these results, ROC analysis showed an excellent diagnostic performance of the LPI, that correctly identified all RAO cases (Area Under the Curve, AUC = 1.0; *p* < 0.001), with an optimal diagnostic cutoff at 0.2 RPI. Conclusions: LPI is a reliable diagnostic technique for RAO, offering the advantages of being quick and simple to perform.

## 1. Introduction

Transradial arterial access (TRA) is a safe and effective alternative to transfemoral access (TFA) for coronary catheterization. It is associated with a significant reduction in access site bleeding, shorter hospital stays, reduced costs, and better outcomes [1,2,3,4,5]. Hence, despite initial concerns about its safety during the learning curve, the use of TRA became progressively widespread [6,7]. Preferential use of TRA is currently recommended by practice guidelines, especially in the setting of acute myocardial infarction (AMI) [3,4,8,9]. On the other hand, clinically relevant complications have been reported after TRA [10,11,12,13,14]. Among these, Radial Artery Occlusion (RAO) is the most frequent, ranging from <1% to 33% of cases, depending on the specific population, procedural characteristics, and the diagnostic method [11,13,14]. Despite being mostly asymptomatic, RAO is not a benign event and deserves clinical attention [10,11,15,16]. However, diagnosis of post-procedural RAO is often missed, also due to demanding diagnostic examination [17].

We recently described a novel noninvasive diagnostic test for RAO, using the Laser Perfusion Imaging (LPI) [16]. In brief, a low-power laser beam generates color-coded maps of hand perfusion, allowing a quick and easy, operator-independent, non-invasive diagnosis of RAO.

The aim of the present study was to evaluate the diagnostic performance of the LPI for the detection of post-procedural RAO compared to vascular duplex examination, in a series of consecutive patients undergoing coronary procedures through the TRA.

## 2. Experimental Section

### 2.1. Patient Selection

Consecutive patients undergoing elective coronary catheterization through a left trans-radial approach at the Magna Graecia University were included.

Exclusion criteria were age <18 or >90, recent TRA, recent AMI or percutaneous coronary intervention (PCI), active malignancy, autoimmune disease, GFR <30 mL/min or dialysis, liver failure, respiratory failure, bleeding disorders, recent stroke, functional impairment of the left arm, pregnancy, severe anemia or thrombocytopenia.

The study protocol was approved by the local ethics committee and all patients provided a written informed consent.

### 2.2. Clinical Evaluation

Before coronary angiography, the patients underwent a complete physical examination, including the following: The *Allen test*, performed as previously described [18,19]; the *reverse Allen test* (rAT) [20]; the Barbeau, performed using a pulse-oximeter, as previously shown [20,21,22]; the *reverse Barbeau* (rBT), with a type D response considered indicative of arterial occlusion [20]. These clinical tests were repeated 24–48 h after radial artery catheterization. Local pain was assessed using a visual analogue scale (*VAS*) ranging from 0 (no pain) to 10.

### 2.3. Laser Perfusion Imaging Evaluation

This examination was performed in the morning before TRA and repeated 24–48 h after catheterization by means of a PeriScan PIM III (Perimed AB, Järfälla, Sweden) Laser Doppler (LD), at 25 °C, after acclimatization, to avoiding any interference by environmental factors. Data were recorded and analysed with Perimed PimSoft (Perimed AB, Järfälla, Sweden).

**Figure 1:**
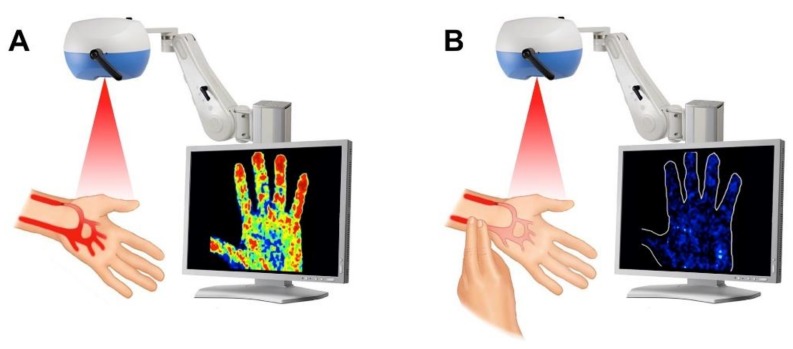
Description of the static Laser Doppler examination (static LDPI test).The static LDPI test was performed taking a color-coded perfusion image of the whole left hand palm in rest condition (**A**) and after ulnar occlusion (**B**).

A Laser Doppler Perfusion Imaging (static LDPI) test was performed taking a color-coded perfusion image of the whole left hand palm in rest condition (Basal Perfusion) and two minutes after ulnar artery occlusion (Radial Perfusion), as we previously described [16]. Diagnosis of RAO was obtained comparing the mean perfusion measured at baseline to that detected after ulnar occlusion (Figure 1). A Radial Perfusion Index (RPI) was calculated as the ratio between the basal radial perfusion and the value measured under ulnar occlusion. LPI operators were blinded to Vascular Duplex (VD) results.

### 2.4. Vascular Duplex

VD, used as the standard of reference, was performed at the same time of the LPI, but operators were blind of the LPI results. A 9 MHz probe (9L-D probe, Vivid E9, GE Healthcare, Wauwatosa, WI, USA) was used. RAO was defined as the absence of flow at VD examination of the radial artery. Two weeks of body-weight-adjusted therapeutic dosing of Low Molecular Weight Heparin (LMWH) was prescribed to patients with RAO to favor recanalization.

### 2.5. Coronary Angiography

Radial artery catheterization was performed as previously described, using 25 cm-long 6F sheaths (Terumo Corporation, Tokyo, Japan) [23]. The left radial artery was used as the default access, for its safety profile [24]. Puncture and cannulation of the radial artery was defined as “challenging” in the following cases: Multiple (≥3) punctures were needed; upgrade to a different needle was needed; need to further attempt by a second physician. Procedural time was measured from the positioning of the sterile towel to sheath removal.

### 2.6. Periprocedural Antithrombotic Treatment

A periprocedural fixed dose of 3000 units of Unfractionated Heparin (UFH) was used. In case of percutaneous coronary intervention, a total fixed dose of 5000 IU of UFH was administered and the Activating Clotting Time (ACT) was monitored during the procedure to maintain a target ACT of 250–300 s. In addition, an intravenous ASA loading dose of 250 mg was administered in case of PCI. Antiplatelet agents were managed as clinically indicated. TRA coronary angiography was allowed both on single- (SAPT) and dual-antiplatelet treatment (DAPT), while DAPT was required for PCI.

### 2.7. Hemostasis

Arterial sheaths were removed at the end of the procedure and a TR Band (Terumo) was applied. Patent hemostasis was confirmed through rBT. The band was checked hourly, with gradual pressure release, until complete deflation, followed by a 5′ observation. In absence of spontaneous bleeding, the compression device was then removed, and a sterile non-compressive banding was applied to cover the wound. During the removal procedure, motor function, vascularization and neurologic sensitivity at the access site were checked.

### 2.8. Statistical Analysis

Receiver operating characteristic (ROC) curves were used to evaluate the diagnostic performance of LPI, as previously described [23]. Comparison of discrete variables between the study groups was performed using the Pearson’s *X*^2^ test, while continuous variables were compared using the Mann-Whitney U test. Paired comparisons were performed with the Wilcoxon signed-rank test, as already previously described [25]. A *p <* 0.05 was considered statistically significant.

Sample size calculation was based on an expected incidence of RAO of ranging 8%–14%. We calculated that 64 to 100 patients would have been required to reach an 80% power for exclusion of a difference of more than 12% between the two diagnostic methods. All analyses were performed with SPSS version 22 (IBM Statistics, Armonk, NY, USA).

## 3. Results

### 3.1. Study Population

A total of 100 consecutive patients undergoing elective left TRA, 68 males and 32 females, with age ranging 40–87, were included in the study.

A previous TRA had been performed in 18 patients (18%). Most patients were already on dual antiplatelet therapy (DAPT) (Table 1).

A radial pulse was detectable in all patients before the procedure. The basal left hand perfusion was 145 ± 49 perfusion units (PU)s at LDPI, with a modest reduction after ulnar occlusion (129 ± 49 PUs; *p* < 0.001).

### 3.2. Procedural Data

The radial artery was successfully cannulated in all cases. However puncture was challenging in 14% of cases. A radial artery spasm was registered in 7% of procedures, 4 males (5.9%) and 3 females (9.4%), but didn’t result in access failure. Procedural time was significantly prolonged for patients experiencing radial spasm of challenging puncture of the radial artery (*p* < 0.001) and in those undergoing coronary interventions (*p* = 0.013). The TR-Band was kept for 5.3 ± 1.5 h (5.4 ± 1.7 in male; 5.2 ± 1.2 in female). Significant (*VAS* > 5) local pain was reported by 10 patients, 5 males (7.4%) and 5 females (15.6%). Analgesics were needed in 2 patients to reduce symptoms (Table 2).

### 3.3. Post-Procedure Evaluation

The incidence of post-procedural RAO at vascular duplex scan (VDS) was 9% in the overall population, 7.4% in men and 12.5% in women. A palpable radial pulse was present in 92% of patients. Of note, examination of the radial pulse yielded a sensitivity of 56%, a specificity of 97%, a Positive Predictive Value (PPV) of 63% and a Negative Predictive Value (NPV) of 96% for assessment of radial patency.

The reverse Allen Test (rAT) was pathologic in 12 patients (13.2%) with patent radial artery and in 7 (77.8%) patients with RAO (sensitivity = 78%, specificity = 87%, PPV = 37%, NPV = 98%). Among patients with patent radial artery, 85 (93%) had a normal rBT, while 8 (89%) had a pathological rBT in the RAO group (sensitivity = 89%, specificity = 93%, PPV = 57%, NPV = 98%).

### 3.4. Characterization of Patients with Post-Procedural RAO

No significant baseline differences were registered between RAO patients and those with patent radial artery, except for DAPT that was more frequent among RAO patients (*p* = 0.044), and Mean Platelets Volume (MPV), that was lower in the RAO group (*p* < 0.05).

RAO patients had kept the hemostatic band significantly longer (*p * < 0.01) and reported more severe access site pain (*p* < 0.05).

### 3.5. Laser Perfusion Examination

**Figure 2:**
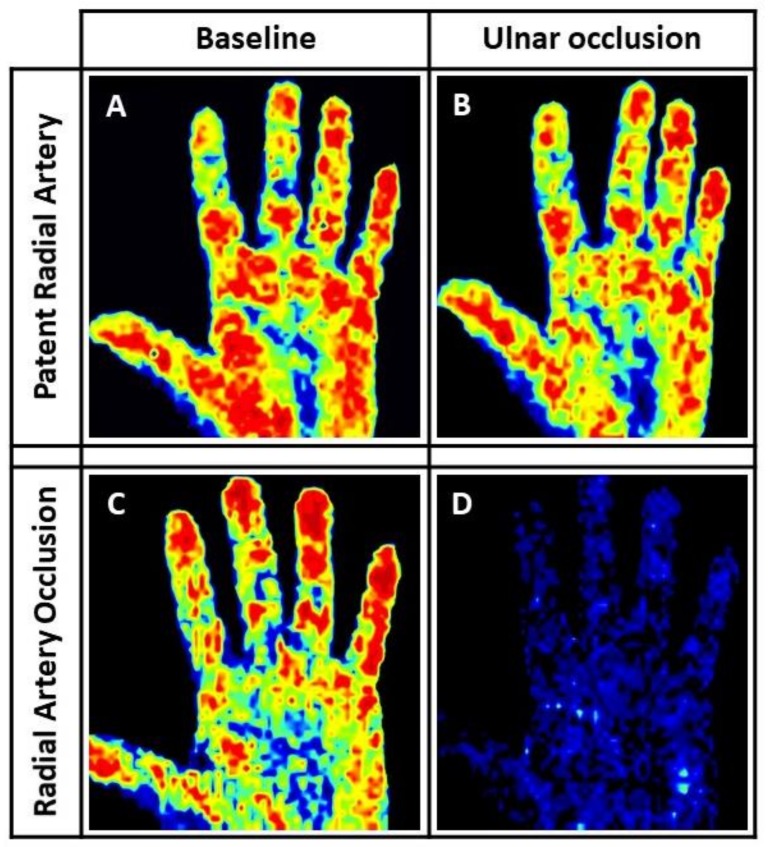
Laser Perfusion Imaging (LPI) of Radial Artery Occlusion (RAO). (**A**) resting left hand LDPI. (**B**) left hand LDPI during complete manual ulnar artery occlusion in a patient with patent radial artery, showing no substantial difference compared to the basal scan (color coded). Accordingly, the RPI was 0.95 in this case. (**C**) resting left hand LDPI. (**D**) left hand LDPI during complete manual ulnar artery occlusion in a patient with RAO, showing ominous reduction of hand perfusion (“blue hand” sign). Consistently with the visual assessment, a dramatic drop of the RPI to 0.17 (82% reduction compared to basal value) was registered in this patient after the trans-radial access (TRA) procedure.

The method for detection of RAO is described in Figure 2. The day after the transradial procedure, left hand basal perfusion was similar to the pre-procedural value (147 ± 46 PUs, *p* = 0.581), while mean perfusion after ulnar occlusion was modestly lower (119 ± 53 PUs, *p* = 0.145). Similarly, no significant changes were observed among RAO patients (125 ± 61 vs. 133 ± 43; *p* = 0.575). On the other hand, a significant post-procedural reduction in mean perfusion under ulnar occlusion was observed in these patients (19 ± 7 vs. 107 ± 63; *p* = 0.008).

**Figure 3:**
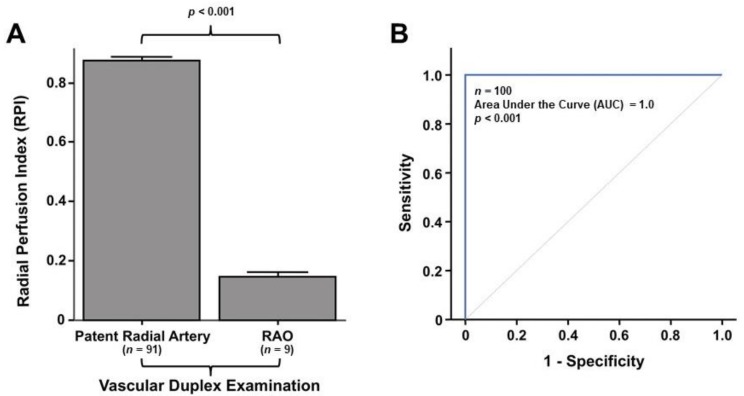
Post-procedural measurement and diagnostic performance of the Radial Perfusion Index (RPI) for diagnosis of RAO. (**A**) The error bars depict the mean RPI measured 24–48 h after the procedure in patients with patent Radial Artery (left) and in patients with RAO (right). (**B**) ROC analysis showed an excellent diagnostic performance of the LPI in the present study, with an Area under the Curve (AUC) of 1.

Accordingly, the mean post-procedural RPI was 0.15 ± 0.04 in patients with RAO and 0.89 ± 0.13 in patients without RAO (*p <* 0.001) (Figure 3A). Similar results were obtained when the RPI was normalized by the pre-procedural measurement (16.9% vs. 99.7%; *p <* 0.001). In line with these results, ROC analysis showed an excellent diagnostic performance for the LPI (Figure 3B) (AUC = 1.0; *p <* 0.001). Using the diagnostic cutoff of 20% (<0.2 RPI), the LPI identified RAO with a sensitivity and a specificity of 100%. No differences were found in the diagnostic performance between males and females at ROC curve analysis.

**Figure 4:**
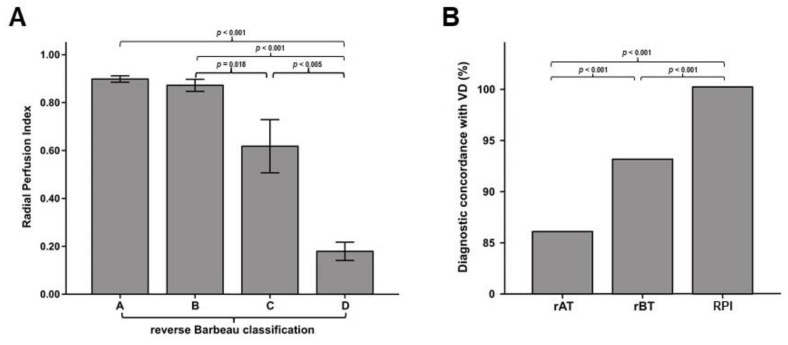
Quantitative value of the RPI. (**A**) The error bars depict mean RPI across different classes ot the reverse Barbeau Test (rBT). (**B**) The bar graph shows diagnostic concordance with the Vascular Duplex (VD), the standard of reference. Diagnostic concordance was significantly and progressively increased from the reverse Allen Test (rAT) (86%), through the rBT (93%) to the RPI (100%).

RPI measurement provided a quantitative estimation of left hand perfusion. In fact, the mean RPI was progressively lower from class A through class D of the rBT (*p <* 0.001), with larger breakdowns between class B and class C (*p* = 0.018) and between class C and class D (*p* = 0.006) (Figure 4A). The diagnostic concordance with the standard of reference (vascular duplex) was 86% for the rAT, but was progressively increased with the rBT (93%; *p <* 0.001 vs. rAT) or the LD-based RPI (100%; *p <* 0.001 vs. the rAT or vs. the rBT) (Figure 4B).

## 4. Discussion

The major finding of the present study is that the Laser Perfusion Imaging, a quick and simple operator-independent diagnostic test, efficiently detects radial artery patency after catheterization.

The availability of a simple and operator-independent test for RAO has a large potential. RAO is most often asymptomatic. However, an occluded radial artery cannot be the access site for successive percutaneous procedures, it cannot be used as arterial graft for coronary bypass. Furthermore, RAO limits ipsilateral ulnar access. However, the frequent absence of symptoms is not the only reason why RAO often remains undiagnosed. In fact, most centers perform no routine evaluation of the radial artery after TRA [17]. Reasons for this include the need for experienced sonographers, given that simple objective pulse examination is not sufficient to detect RAO. In fact, in a recent study, 2% of patients had no radial pulse and radial artery occlusion was found in 9% of patients at color-Doppler [26]. In line with these results, in the present study only 97% of patients with a patent radial artery and as much as 44% of patients with post-procedural RAO had a palpable radial pulse, yielding a low positive predictive value. Although better than pulse examination, neither the rAT nor the rBT achieved a high degree of concordance with the duplex examination. On the contrary, LPI allowed early recognition of post-procedural RAO. This finding has a relevant clinical impact, as prompt treatment is often effective to achieve radial artery recanalization and to maintain long-term patency. As the pathophysiology of early post-procedural RAO is most often thrombotic, anticoagulation is often able to achieve recanalization [6]. In fact, back in 2010 a single center observational study on post-procedural RAO reported a high recanalization rate (86.7%) in patients who had been treated with low molecular weight heparin (LMWH) for four weeks after the percutaneous procedure, as compared to a 19.1% recanalization rate in patients not receiving an anticoagulation therapy [27]. More recently, Bernat and colleagues performed immediate 1-h ipsilateral ulnar artery compression in patients experiencing RAO, in an attempt to reopen the radial artery by increasing peak velocity blood flow [28]. Although a substantial reduction in RAO from 2.9% to 0.8% after ulnar artery compression was observed, the study wasn’t placebo controlled and the patients were not randomly assigned to the promising treatment. On the contrary, all patients diagnosed with RAO underwent ulnar artery compression, the study wasn’t placebo controlled and the patients were not randomly assigned to the promising treatment. On the contrary, all patients diagnosed with RAO underwent ulnar artery compression [28]. More recently, two larger studies independently confirmed that radial occlusion can be easily and safely treated with ipsilateral ulnar compression [29,30].

Finally, the LPI is operator-independent and provides an objective and reproducible quantitative perfusion value. This might be an advantage, especially in those cases when the presence of anatomical variants or subversions resulting from repeated punctures, inflammatory edema or hematomas, make the duplex examination more difficult. Furthermore, since the LPI doesn’t require a direct contact with the skin. Hence, lack of direct contact of the probe with the skin wound at the puncture site avoids painful reactions and reduces the risk of access site infection [12,13].

Furthermore, the data reported also provide additional evidence on the incidence and predictors of RAO after TRA through the left radial artery (LRA). In fact, LRA access is largely underrepresented in studies on RAO. To this regard, our finding that longer TR-band time was significantly associated with RAO is worth noting. In fact, this finding is in line with previous studies [31,32,33,34]. Interestingly, Lavi et al. reported that shortening the haemostasis time to one hour is able to further reduce RAO incidence [35]. On the contrary, they demonstrated that a further reduction to thirty minutes has no impact on RAO [35]. Since RAO is usually a thrombotic event, which is often related to the presence of micro-dissections, it is tempting to speculate that the association with occlusion time reflects a higher propensity to arterial thrombosis associated with prolonged flow impairment. Furthermore, our finding that post-procedural access site pain was also a predictor of RAO is in line with previous evidence [36], suggesting that prolonged pain after sheath removal might reflect extensive structural damage, which could in turn be a triggering for both acute thrombosis or more pronounced adverse vascular remodeling, thus leading to an increased RAO rate.

Despite the promising results, some potential limitations of this diagnostic technique should be acknowledged. First, LPI could become difficult in case of increased skin thickness. Second, anatomical variations could potentially influence the test. However, in the present study, some cases of anterior interosseous were included but never represented a real threat for the test. Current costs for the LD equipment using in this study are still high, however a simpler and cheaper diagnostic device for assessment of RAO with the LD is currently being developed.

## 5. Conclusions

Results of the present study demonstrate for the first time that the LPI is a reliable test for a quick, easy and operator-independent diagnosis of RAO. The lack of direct contact of the probe with the skin is a further interesting characteristics.

## Figures and Tables

**Table 1:** Baseline patients’ characteristics according to radial artery patency status.

	Total (*n* = 100)	Occluded (*n* = 9)	Not Occluded (*n* = 91)	*p* *
Female	32%	4 (44.0%)	28 (31.0%)	0.40
Age (years, mean ± SD)	66.8 ± 11.4	73.9 ± 11.7	66.1 ± 11.2	0.051
Family history of CAD	30%	2 (22.2%)	28 (30.8%)	0.59
BMI (kg/m^2^, mean ± SD)	27.9 ± 4.4	25.7 ± 3.3	28.2 ± 4.4	0.11
Hypertension	84%	7 (77.8%)	77 (84.6%)	0.59
Dyslipidaemia	51%	4 (44.4%)	47 (51.6%)	0.68
Diabetes	29%	1 (11.1%)	28 (30.8%)	0.22
Smokers	25%	2 (22.2%)	23 (25.3%)	0.84
Previous PCI	26%	3 (33.3%)	23 (25.3%)	0.60
Previous trans-radial access (>3 months)	18%	2 (22.2%)	16 (17.6%)	0.73
Atrial Fibrillation	12%	1 (11.1%)	11 (12.1%)	0.93
LVEF (%, mean ± SD)	51.5 ± 8.2	52.7 ± 6.9	51.4 ± 8.3	0.65
NYHA Class II-IV	61%	6 (66.7%)	55 (60.4%)	0.72
anti-P2Y12 loading dose	15%	1 (11.1%)	14 (15.4%)	0.73
DAPT (Cath-lab)	71%	9 (100.0%)	62 (68.1%)	0.044
ACE-Is/ARBs	75%	6 (66.7%)	69 (77.5%)	0.46
Beta Blockers	58%	5 (55.6%)	53 (59.6%)	0.82
Nitrate	23%	3 (33.3%)	20 (22.5%)	0.46
Oral Hypoglycemics	22%	1 (11.1%)	21 (23.1%)	0.41
Insulin	6%	0 (0.0%)	6 (6.6%)	0.43
Statin	73%	6 (66.7%)	67 (73.6%)	0.65
GFR (mL/min, mean ± SD)	84.2 ± 32.7	69.8 ± 30.6	85.7 ± 32.7	0.17
Hemoglobin (g/dL, mean ± SD)	13.7 ± 1.59	13.2 ± 1.28	13.8 ± 1.61	0.29
Hematocrit (%, mean ± SD)	42.0 ± 4.3	41.1 ± 4.6	42.1 ± 4.3	0.50
Platelets (×10^3^/μL, mean ± SD)	211 ± 54	242 ± 52	208 ± 54	0.068
MPV (fL, mean ± SD)	8.1 ± 1.0	7.4 ± 0.6	8.2 ± 1.0	0.035
Glycemia (mg/dL, mean ± SD)	122 ± 45	109 ± 18	122 ± 47	0.41
Total Cholesterol (mg/dL, mean ± SD)	172.0 ± 36.9	158.1 ± 31.9	173.3 ± 37.2	0.24
LDL Cholesterol (mg/dL, mean ± SD)	108.4 ± 32.4	95.8 ± 29.9	109.7 ± 32.5	0.22
HDL Cholesterol (mg/dL, mean ± SD)	50.4 ± 18.2	49.9 ± 12.6	50.5 ± 18.7	0.93
Triglycerides (mg/dL, mean ± SD)	143.3 ± 79.8	114.8 ± 54.2	146.1 ± 81.5	0.26
Creatinine (mg/dL, mean ± SD)	0.93 ± 0.22	0.93 ± 0.28	0.93 ± 0.22	0.98

Abbreviations: ACE-Is, Angiotensin Converting Enzyme inhibitors; ARBs, Angiotensin Receptor Antagonist; BMI, Body Mass Index; CAD, Coronary Artery Disease; DAPT, Dual AntiPlatelet Therapy; GFR, Glomerular Filtration Rate; HDL, High Density Lipoproteins; LDL, Low Density Lipoproteins; LVEF, Left Ventricle Ejection Fraction; MPV, Mean Platelets Volume; NYHA, New York Heart Association; PCI, Percutaneous Coronary Intervention; SD, Standard Deviation. * *p* value of comparison between “Occluded” and “Not occluded”.

**Table 2:** Patients’ procedural characteristics according to radial artery patency status.

	Total (*n* = 100)	Occluded (*n* = 9)	Not Occluded (*n* = 91)	*p* *
Procedural time (min, mean ± SD)	51.0 ± 32.8	70.6 ± 38.6	49.0 ± 31.7	0.060
Access complications				
Challenging access (puncture)	14%	2 (22.2%)	12 (13.2%)	0.46
Spasm	7%	1 (11.1%)	6 (6.6%)	0.61
Dissection	0%	0 (0.0%)	0 (0.0%)	-
PCI performed	21%	3 (33.3%)	18 (19.8%)	0.34
Nitroglicerin i.a.	21%	3 (33.3%)	18 (19.8%)	0.34
UFH (IUs, mean ± SD)	3530 ± 893	3666 ± 1000	3516 ± 886	0.63
TR-band time (h, mean ± SD)	5.32 ± 1.52	6.67 ± 2.21	5.18 ± 1.38	0.004
Symptoms				
Transient paresthesia	23%	2 (22.2%)	21 (23.1%)	0.95
Local pain (*VAS*, mean ± SD)	0.92 ± 1.80	2.33 ± 2.96	0.78 ± 1.60	0.013
Local pain (*VAS* ≥ 5)	10%	3 (33.3%)	7 (7.7%)	0.014
Banding (post TR-band removal)				
None	35%	1 (11.1%)	34 (37.4%)	0.12
Non compressive	49%	5 (55.6%)	44 (48.4%)	0.68
Compressive Banding	16%	3 (33.3%)	13 (14.3%)	0.14

Abbreviations: i.a., intra-arterial; PCI, Percutaneous Coronary Intervention; TR-Band, trans-radial band; UFH, UnFractionated Heparin; *VAS*, Visual Analogic Scale. * *p* value of comparison between “Occluded” and “Not occluded”.

## Supplementary Materials

The following are available online at http://www.mdpi.com/2077-0383/7/10/319/s1, Figure S1: Study procedure timeline. The flowchart illustrates study procedures in a timeline.
