# Supplementary figures and images for: Hand Laser Perfusion Imaging to Assess Radial Artery Patency: A Pilot Study
Source: J Clin Med. 2018 Oct 2;7(10):319. doi: 10.3390/jcm7100319 (PMC6210442; doi:10.3390/jcm7100319)

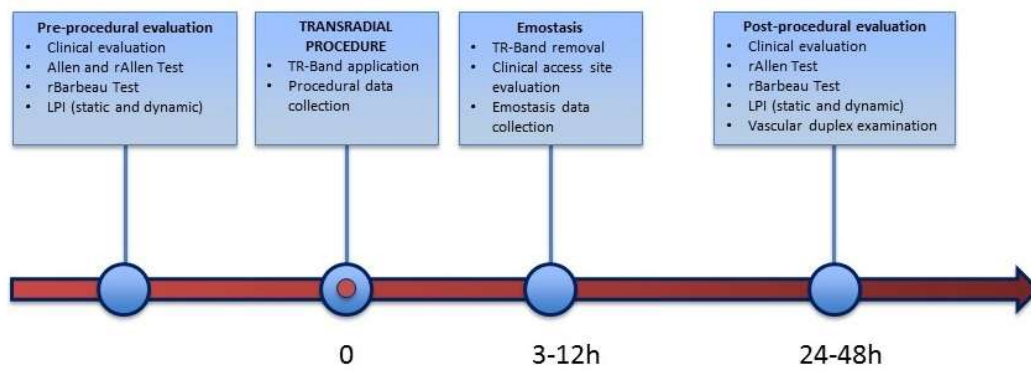

**Figure S1.** Study procedure timeline. The flowchart illustrates study procedures in a timeline.

Supplement: Supplementary file 1 [file jcm-07-00319-s001.pdf]
